# Rapid Classification and Treatment Algorithm of Cardiogenic Shock Complicating Acute Coronary Syndromes: The SAVE ACS Classification

**DOI:** 10.1155/2022/9948515

**Published:** 2022-01-12

**Authors:** Vasileios Panoulas, Charles Ilsley

**Affiliations:** ^1^Department of Cardiology, Royal Brompton and Harefield Hospitals, Guy's and St Thomas' NHS Foundation Trust, Harefield, UK; ^2^Cardiovascular Sciences, National Heart and Lung Institute, Imperial College London, London, UK

## Abstract

**Introduction:**

We aimed to identify the independent “frontline” predictors of 30-day mortality in patients with acute coronary syndromes (ACS) and propose a rapid cardiogenic shock (CS) classification and management pathway.

**Materials and Methods:**

From 2011 to 2019, a total of 11439 incident ACS patients were treated in our institution. Forward conditional logistic regression analysis was performed to determine the “frontline” predictors of 30 day mortality. The *C*-statistic assessed the discriminatory power of the model. As a validation cohort, we used 431 incident ACS patients admitted from January 1, 2020, to July 20, 2020.

**Results:**

Independent predictors of 30-day mortality included age (OR 1.05; 95% CI 1.04 to 1.07, *p* < 0.001), intubation (OR 7.4; 95% CI 4.3 to 12.74, *p* < 0.001), LV systolic impairment (OR _severe_vs_normal_ 1.98; 95% CI 1.14 to 3.42, *p*=0.015, OR _moderate_vs_normal_ 1.84; 95% CI 1.09 to 3.1, *p*=0.022), serum lactate (OR 1.25; 95% CI 1.12 to 1.41, *p* < 0.001), base excess (OR 1.1; 95% CI 1.04 to 1.07, *p* < 0.001), and systolic blood pressure (OR 0.99; 95% CI 0.982 to 0.999, *p*=0.024). The model discrimination was excellent with an area under the curve (AUC) of 0.879 (0.851 to 0.908) (*p* < 0.001). Based on these predictors, we created the SAVE (SBP, Arterial blood gas, and left Ventricular Ejection fraction) ACS classification, which showed good discrimination for 30-day AUC 0.814 (0.782 to 0.845) and long-term mortality (*p*_log−rank_ < 0.001). A similar AUC was demonstrated in the validation cohort (AUC 0.815).

**Conclusions:**

In the current study, we introduce a rapid way of classifying CS using frontline parameters. The SAVE ACS classification could allow for future randomized studies to explore the benefit of mechanical circulatory support in different CS stages in ACS patients.

## 1. Introduction

Cardiogenic shock (CS) is often encountered by frontline cardiac catheterization teams, which are part of primary percutaneous coronary intervention (PPCI) pathways [1]. Even though the aetiology of CS can vary, left ventricular (LV) failure following acute myocardial infarction (MI) remains the most frequent cause accounting for over 40% of cases [2]. Mechanical complications after MI (ventricular septal rupture, free wall rupture, and acute severe mitral regurgitation) in late presenters or patients that have not been revascularized represent a small proportion of CS cases yet carry considerable morbidity and mortality. [3] CS can also be attributed to non-MI causes, such as decompensated chronic heart failure (often dilated cardiomyopathy), valvular heart disease, myocarditis, stress-induced cardiomyopathy (Takotsubo syndrome), or arrhythmias (atrial or ventricular) [4].

Despite the significant advances in reperfusion therapy and percutaneous mechanical circulatory support (MCS) devices, mortality among patients presenting with CS remains very high, ranging from 25% to 50% [5, 6]. In a recent large cohort of 21,210 patients in London with ST segment elevation myocardial infarction (STEMI), CS was observed in 8.9% of patients with the incidence increasing over time and high mortality of 45%–70% [1]. It should, however, be mentioned that the definition of shock in these databases remains dubious due to the lack of detailed echocardiographic, hemodynamic, and biochemical parameters.

Traditional definitions of cardiogenic shock in major cardiac societies [4] and randomized studies [2] have included a systolic blood pressure of <90 mmHg (or requiring inotropes to keep SBP ≥90 mmHg) for 30 min or more and evidence of impaired organ perfusion (on clinical examination, lactate >2.0 mmol/L and urine output<30 mls/h). Recently, Baran et al. [7] published an easy-to-remember classification of cardiogenic shock based on clinical, observational, biochemical, and hemodynamic parameters. Even though it is a truly thorough and conceptually easy classification, its extensive definitions, including hemodynamic parameters, render its prompt applicability in the frontline settings difficult. Furthermore, the lack of bedside echocardiographic evaluation is surprising given that often LV systolic impairment precedes SBP drop in acute MI settings and is an independent predictor of outcome in CS patients [8].

Developing an immediate response algorithm (within 5 min), which includes early MCS, is important given that registry evidence suggests improved survival when left ventricular support is initiated early on [9].

In the current study, we aimed to identify the independent “frontline” predictors of 30-day mortality in a large cohort of patients presenting with ACS and create a straightforward ACS cardiogenic shock classification that could assist physicians in decision making for early MCS deployment [2].

## 2. Methods

From January 2011 to December 2019, a total of 17908 PCIs were performed in Harefield Hospital (Royal Brompton and Harefield NHS Foundation Trust, London, UK). Of those, 12458 were in the context of ACS, whereas 11,439 were incident presentations within this time frame, excluding those ones with mechanical complications. As a validation cohort, we used 431 incident ACS patients undergoing PCI in our institution from January 1, 2020, to July 20, 2020.

Routine clinical data on demographics, clinical characteristics on presentation, admission observations, electrocardiogram, bedside LV function (prior to procedure), arterial blood gas data (on admission, prior to procedure), past medical history, and procedural characteristics were prospectively collected. Cardiac arrest was classified as follows: EMD (electromechanical dissociation), asystole, VT (ventricular tachycardia), VF (ventricular fibrillation), or unknown.

All patients from the derivation cohort were followed up from the time of the procedure until death or censored 01.06.2020. The primary outcome was 30-day mortality. Vital status was ascertained using the National Patient Demographic Service, which incorporates National Death Registry information as well as local notifications. All patients from the validation cohort were followed up from the time of the procedure until death or censored 30.08.2020.

### 2.1. Ethics

The current study complies with the Declaration of Helsinki. Following consultation with our local research ethics committee, no informed consent was required as all data were retrospective, anonymized, and part of an ongoing audit.

### 2.2. Statistics

All continuous variables were tested for normality using the Kolmogorov–Smirnov test. Data are presented as percentages, mean ± standard deviation (SD), or median (interquartile range). Differences in proportions were tested with Chi-square test or Fisher's exact test, and differences in continuous variables were tested with ANOVA/Student's *t*-test or Kruskal–Wallis/Wilcoxon's signed-rank sum test for parametric and nonparametric variables, respectively.

We deliberately elected to create a 30-day mortality prediction model based on admission information and non-past medical history, which can often be inaccurate or impossible to obtain at the frontline when patients are confused, in shock, in pain, or intubated and ventilated.

Forward conditional logistic regression analysis was performed to determine the main “frontline” predictors of 30-day mortality. All categorical variables that were significantly different in the two groups (alive vs. deceased at 30 days) and all continuous/ordinal variables that were significantly different *p* < 0.05 with an area under the curve AUC > 0.7 for 30-day mortality were included in the final binary logistic regression model.

For continuous variables of interest (lactate), we used the Youden's index [10] to identify optimal cutoff points on the receiver operating characteristic (ROC) curve.

The binary logistic regression model (dependent variable 30-day survival) included the following independent variables: age, gender, systolic blood pressure (SBP), ECG changes, bedside LV systolic assessment, arterial lactate, pH, base excess (BE), cardiac arrest, ongoing resuscitation, and intubation-ventilation preprocedure.

The *C*-statistic-related AUC of the models created was compared using the DeLong methodology [11].

The selected variables included in the final model were also included in a Cox-regression model to assess whether they are predictors of long-term mortality.

Based on the binary logistic regression model (Table 1) and currently existing cardiogenic shock protocols, the simplified SAVE (Systolic blood pressure, Arterial blood gas, and left Ventricular Ejection fraction) ACS A, B, C, D, and E shock groups were defined (Table 2), and their classification power, for 30-day mortality, was tested using ROC curves.

In the validation cohort, we assessed the *C*-statistic of Model 6 (Table 3) and the *C*-statistic of the SAVE ACS classification in predicting 30-day mortality.

## 3. Results

During the study period, a total of 11439 index ACS cases that underwent PCI were included in the derivation cohort. Of those, 6039 (52.8%) were STEMIs, 5392 (47.1%) were NSTEMIs, and 8 (0.1%) unstable angina cases. Mean age was 64.1 ± 14.5 years and 8280 (72.4%) were male.

Table 4 shows the differences in demographics and frontline data based on vitality status at 30 days. Patients who subsequently died in 30 days were older, most commonly females, with a higher prevalence of clinical hypoperfusion and pulmonary oedema on arrival, lower systolic blood pressure, and a higher incidence of ECG changes (particularly T-wave inversion and ST depression)

A total of 3752 patients (32.8%) had arterial blood gas (ABG) prior to any intervention due to concerns raised in clinical assessment (Table 4). Indeed, patients who had their lactate measured had significantly higher 30-day mortality compared to those who did not have an ABG (17.1% vs. 3.3%, *p* < 0.001)

In patients who had their lactate measured, 30-day mortality AUC for admission lactate was 0.780 (0.759 to 0.801) (*p* < 0.001). Applying the Youden index, the optimal lactate cutoff value to predict 30-day mortality was 2.65 with a sensitivity of 0.646 and a specificity of 0.796.

Patients who were deceased at 30 days exhibited significantly worse LV function on admission on bedside echocardiography. Cardiac arrest was present in more than half (51.4%) of patients who subsequently died within 30 days, whereas nearly a third (32.1%) were intubated on arrival. Asystole and electromechanical dissociation (EMD) were arrest rhythms that were most likely to predict death at 30 days.

In Table 5, we present the past medical history and procedural data based on vitality status at 30 days.

**Figure 1:**
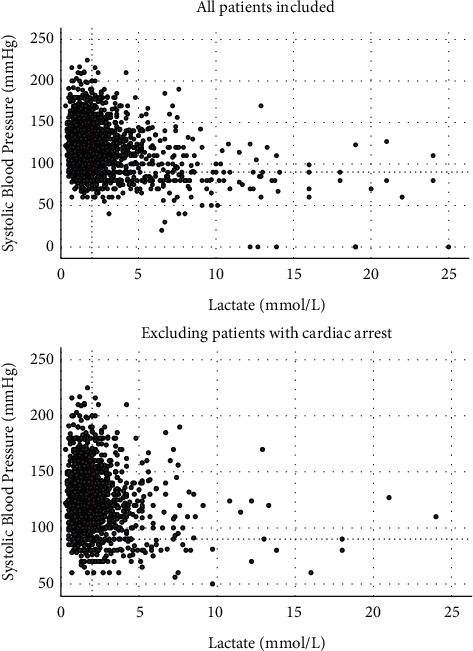
Scatter plots of lactate and systolic blood pressure (SBP) of patients presenting with acute coronary syndromes. In the top panel, all patients are included, whereas in the bottom one, patients with previous or ongoing cardiac arrest are excluded. The red dotted line indicates lactate cutoff of 2 mmol/L, whereas the blue dotted line indicates the traditional 90 mmHg SBP.

Figure 1 demonstrates the presence of hypoperfusion (lactate >2 mmol/L) in a third of normotensive ACS patients (29.2% of patients with SBP >90 mmHg have a lactate between 2 and 5 mmol/L, whereas 5.7% of normotensive patients have a lactate >5 mmol/L). Even when excluding patients with resuscitated cardiac arrest, 30% of normotensive ACS present with a lactate of >2 mmol/L.

When examining hypoperfused patients (defined as lactate >2 mmol/L), 78% had SBP >90 mmHg.

### 3.1. Multivariable Analysis

A total of 892 patients were included in the multivariable analysis (full set of data) as shown in Table 3.

**Figure 2:**
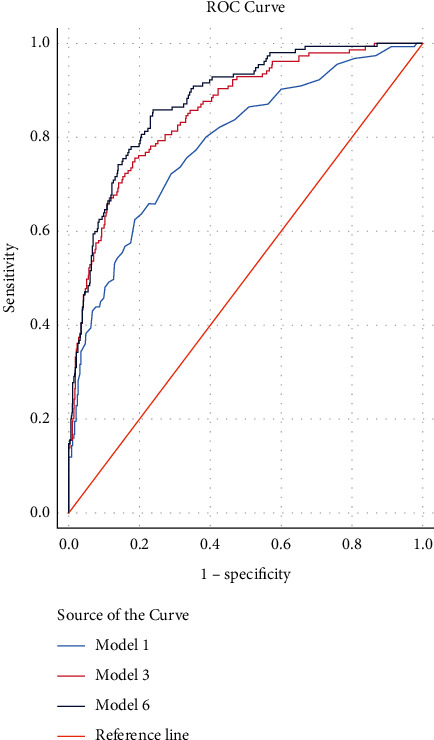
Comparison of the receiver operating characteristic (ROC) curves of different regression models predicting 30-day mortality. Model 1: Lactate. Model 3: lactate, intubation, age. Model 6: lactate, intubation, age, base excess, left ventricular ejection fraction, and systolic blood pressure.

Independent predictors of 30-day mortality by order of significance were lactate, intubation/ventilation on arrival, age, BE, LVEF category, and SBP. The different models and corresponding *C*-statistics are shown in Table 3. In Model 6, an AUC of 0.879 (0.851 to 0.908) was achieved, which suggests a very good performance (Figure 2). As noted, the contribution of SBP to the incremental value of the *C*-statistic is rather small, and no statistically significant difference was seen when comparing the *C*-statistic of Models 5 and 6.

In Supplementary Table 1, all variables included in Model 6, except for SBP, predicted long-term survival.

When using the same variables but dichotomized as per established criteria [7] (e.g., SBP <90 mmHg, lactate cutoff values of 2 and 5(7), and BE tertiles), the model discrimination remains excellent with an AUC of 0.883 for 30-day mortality (Table 1).

In the validation cohort, Model 6 variables demonstrated an excellent discrimination for 30-day mortality with a *C*-statistic of 0.949 (0.887–1) (*p* < 0.001).

**Figure 3:**
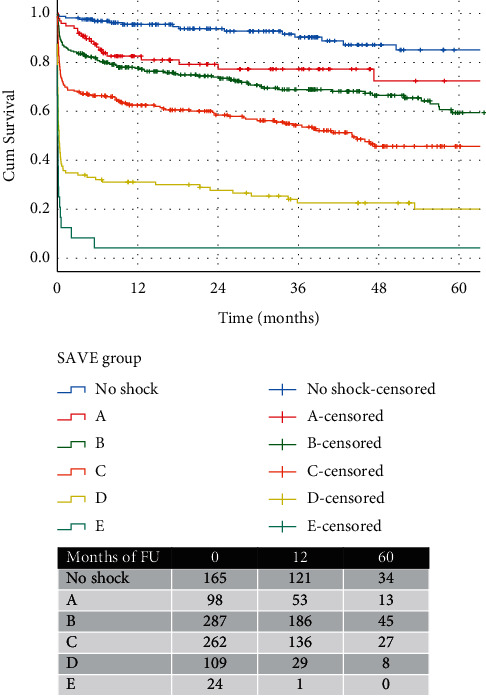
Kaplan–Meier curves showing 5-year survival of patients in different stages of shock, as per SAVE ACS classification.

Frontline SAVE (systolic blood pressure, arterial blood gas, and left ventricular ejection fraction) ACS shock classification: the frontline shock classification includes only 4 variables readily available within a short time frame from admission; arterial SBP, LV function, lactate, and BE. Since age and intubation, even though strong predictors of mortality are not indicative of the hemodynamic status of the patients, we elected to use the 4 remaining variables (from Table 1) to classify patients in the following subgroups (no shock, A, B, C, D, and E). Each group can subsequently predict 30-day and long-term mortality (Table 2, Figure 3).

It should be noted that this classification relies more on the variables that were shown to significantly contribute to the forward conditional regression analysis from Table 3. Less importance was given to systolic blood pressure in defining shock as it contributed nonsignificant increments in AUC in Model 6 (Table 3).

The SAVE ACS classification AUC for 30-day mortality was 0.814 (0.782 to 0.845, *p* < 0.001), suggestive of a very good discrimination. Furthermore, the SAVE ACS classification predicted long-term survival as shown in Figure 3 (*p*_log−rank_ < 0.001).

The discrimination of the SAVE ACS classification in the validation cohort was very good with a *C*-statistic of 0.815 (0.725 to 0.905, *p* < 0.001)

**Figure 4:**
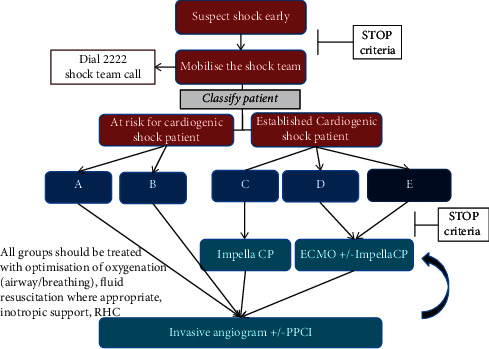
Proposed treatment algorithm for patients presenting with acute coronary syndromes and shock. Early use of mechanical circulatory support is advocated for patients in the C-E groups.

Based on the SAVE ACS classification, the authors created an algorithm for the use of MCS in the patient group with the highest 30-day mortality and biochemical/echocardiographic indices compatible with classic or worsening shock (Figure 4). Prior to any decision for MCS, the shock team would ensure there are no contraindications (Supplementary Figure (available here)).

## 4. Discussion

In the current retrospective study, we have identified 5 variables apart from age, that are readily available to frontline staff (SBP, LV function, intubation, arterial lactate, and base excess) that can help identify rapidly those ACS patients at higher risk of death at 30 days. These variables alone showed an excellent discriminatory power for predicting 30-day mortality with an AUC of 0.883 in the derivation and 0.949 in the validation cohort. Based on these variables, we introduced the “SAVE ACS” classification, which can be rapidly carried out by frontline staff to classify ACS patients in different stages of CS. This would allow physicians to make informed decisions on early MCS use in patients with a higher 30-day mortality risk (Figure 4). The current study also highlights the important concept of “normotensive shock,” originally introduced by Menon et al. [12] in 2000, as nearly 80% of hypoperfused patients (defined as lactate >2 mmol/L) were shown to have a SBP >90 mmHg (Figure 1).

CS definitions have been very variable across different studies. Few decades ago, Forrester et al. in their classification [13] using right-sided heart catheterization described the role of cardiac hemodynamics in stratifying the risk after acute MI in the pre-PPCI era. Patients in Forrester et al.'s subgroup IV with a pulmonary capillary wedge pressure (PCWP) >18 mm Hg and a cardiac index (CI) <2.2 L·min^−1^·m^−2^, indicative of CS, had a mortality of 51%. In the ongoing DANGER trial, tissue hypoperfusion was defined as lactate ≥2.5 mmol/L, persistent hypotension with SBP<100 mmHg, and/or need for inotropes and LV <45% on echocardiography [14]. In the anticipated ECLS-shock trial [15], the entry criteria include, amongst others, SBP<90 mmHg for >30 min or catecholamines to maintain pressure >90 mmHg and an arterial lactate of >3 mmol/L. In another large trial [16], the EURO Shock randomized study entry criteria include yet again hypotension defined as SBP<90 mmHg for at least 30 min or inotropic support to maintain a SBP>90 mmHg and organ hypoperfusion measured as lactate >2 mmoL. One common theme in these studies is the difficulty in recruitment, which can be explained by the exclusion of patients with normotensive hypoperfusion, who constitute a third of ACS patients. As shown in Figure 1, in our cohort, the majority (78%) of hypoperfused patients (lactate >2 mmol/L) have SBP over 90 mmHg. It may also well be that patients who are both hypoperfused and hypotensive are the ones far too deep in the spiral of cardiogenic shock, with futile outcomes despite MCS deployment.

Baran et al. [7] identified these differences in classifying shock in ongoing clinical trials and published a comprehensive consensus on shock classification that would unify the scientific community and lead to more reproducible outcomes. One of the well-thought clauses in the SCAI shock classification for the hemodynamic definition of shock is “>30 mmHg drop from baseline SBP.” This, however, takes for granted the knowledge of the patient's normal baseline SBP, which is often an unknown parameter. Hence, the definition of shock based on SBP drop from baseline can be challenging. Invasive hemodynamics, even though desirable for established intensive care patients, cannot be readily obtained in the primary PCI setting where shorter door-to-balloon [17] times have been associated with improved future survival. Door-to-unload [18] time is also emerging as an important concept and delay in shock classification and timely MCS deployment may have important implications in survival. Hence, studies that advocate physicians to wait for 30 min [15, 16] to assess the presence of shock may be allowing patients to drift deeper into the spiral of shock, depriving them of the benefit of early MCS [19]. Of note, in our dataset, only 15% of patients with lactate between 2 and 5 mmol/L had a SBP <90 mmHg, whereas of those with a lactate >5 mmol/L, only 45% had a SPB <90 mmHg. This suggests that studies advocating a 90 mmHg cutoff for the definition of shock are excluding a vast number of patients who have already exhibited tissue hypoperfusion.

In a multivariable analysis of the FRENCHSHOCK registry (*N* = 772) of patients admitted to ITU with cardiogenic shock [20] (albeit nonischemic in 64%), independent predictors of 30-day mortality included age, low SBP, high arterial lactate, low eGFR, and low LVEF. These results broadly match our set of variables even though our data relates to patients presenting with ACS. The similarity of these studies suggests that cardiogenic shock of any aetiology has similar predictors, assuming attempts for effective revascularization have occurred where indicated. Furthermore, in line with our findings, a study of 165 ischemic cardiogenic shock patients identified baseline serum bicarbonate values to be predictive of 28-day mortality [9]. Metabolic acidosis is a multifactorial event caused by the combination of bicarbonate loss by the kidneys, mounting ketones, accumulation of inorganic acids, and systemic lactic acidosis [21]. Given that acute kidney injury emerges early in patients with CS [22], it is possible that in patients with impending CS, metabolic acidosis ensues first in the pathophysiological cascade, tagging the patients at risk (group B in the SAVE ACS classification). Furthermore, it has been established that acidosis reduces cardiac contractility and enhances vascular hyporesponsiveness to vasopressors, precipitating a vicious circle leading to worsening CS [21].

The importance of arterial lactate value in predicting short-term mortality has been identified in several studies. In patients with septic shock, a presenting blood lactate of more than 2.5 mmol/L showed the best discrimination (the largest area under the ROC curve to predict 28-day mortality of 0.7) [23]. A systematic review of 33 studies [24] concluded that patients presenting to the hospital with a lactate of more than 2.5 mmol/L on admission should be closely monitored for signs of deterioration. Another recent study [25] on an unselected A&E population of 14015 patients, revealed an optimal lactate cutoff (using also Youden's index) of 2.6 and an AUC of 0.711. The 30-day mortality in those with high lactate was 20.8% versus 6.5% in those in the lower lactate group. Interestingly, the ongoing DANGER trial also uses a lactate cutoff of 2.5 based on a retrospective study of 2094 suspected STEMI patients [26]. In the latter study [26], the use of SBP, LVEF, and lactate as continuous variables led to an AUC of 0.88 (*p* < 0.001) for prediction of 30-day mortality, highlighting the importance of including lactate and echocardiographic LV assessment in the definition of shock. In our data, a lactate value of 2.65 was the best discriminator of 30-day mortality and appears to be in agreement with all aforementioned studies.

In patients with MI-related shock, an algorithm from the IABP-SHOCK II trial was produced to predict mortality [27]. These predictors included age >73 (1 point), prior stroke (2 points), glucose on admission>10.6 mmol/L (1 point), creatinine on admission >132.6 (1 point), TIMI flow grade <3 after PCI (2 points), and blood lactate on admission of >5 mmol/L (2 points). Of interest, the predictive value of this score had an AUC of 0.74, which is lower than that the one seen in our model or indeed the one produced by Frydland et al. using only three variables [26]. Furthermore, creatinine is not always readily available on admission nor is the TIMI flow after PCI. Therefore, this algorithm is not fit for this purpose, as it cannot rapidly classify patients into shock groups who may be in need of early MCS before PCI. Similarly, the Global Registry of Acute Coronary Events (GRACE) score [28] was developed for patients presenting with ACS. The GRACE score predicts 6-month and longer-term mortality [29] with *C*-statistics in the 0.8 mark. The GRACE score was later slightly modified by Fox et al. [30] using a cohort of 43810 patients. Of interest, perfusion indices, such as lactate, are not included in the GRACE score, whereas “creatinine” and “troponin,” which are amongst the 8 variables required to calculate the score, are not always readily available when performing an initial assessment of a patient with ACS.

In the current study, we aimed to highlight that in patients presenting with ACS, CS is a continuum and traditional definitions with arbitrary cutoffs of SBP <90 mmHg have to be replaced by more robust, yet easy-to-use, classifications that reflect the severity of tissue hypoperfusion. Patients admitted with ACS are often in pain and emotional distress, leading to increased sympathoadrenal activation [23]. Hence, it is not uncommon that patients with tissue hypoperfusion present with a systolic blood pressure over 90 mmHg, driven purely by the activation of their sympathetic system. Once the stressor is removed (revascularization), coupled with reperfusion [31], the blood pressure starts to drift downwards unmasking the underlying hemodynamic compromise. In such cases, echocardiography, metabolic acidosis, and raised lactate can identify the underlying impending doom at an earlier stage, thus allowing physicians to act faster, preventing the patient from sliding into the irreversible part of the CS spiral.

This, as every retrospective study, is not without its limitations. Our classification was created based on patients who had a full set of variables (selection bias). These were, invariably, patients at the sicker end of the spectrum as demonstrated by the significantly higher 30-day mortality of those who had lactate measured. They, however, are also the patients who would benefit the most from a rapid shock classification and MCS deployment. Heart rate at presentation was not accurately recorded; hence, it was not included in our models. The predictive power of our classification was confirmed in the validation cohort from our institution. Furthermore, we would like to highlight that this classification was derived from ACS patients that underwent PCI, and it excluded those who had normal coronaries, those that underwent emergency coronary artery bypass, or indeed those in whom any intervention was deemed futile. Collaborations with other centers have been set in motion to validate our findings in large independent ACS cohorts. Figure 4 is conceptual and not supported by any data in this or other studies. It is, however, proposing an escalating level of MCS depending on the level of CS the patient is in, with the least invasive option (Impella CP) for the early/classic stages of shock (C) and the use of ECMO in combination with Impella for those in deeper shock (D and E).

A future randomized trial assessing the benefit of early MCS use in SAVE ACS groups C, D, E, and potentially B would allow us to draw conclusions on the benefits of MCS in these groups.

## 5. Conclusions

The current study describes an easy, rapid classification of CS using variables readily available to frontline physicians. The SAVE ACS aims to complement rather than replace the comprehensive, established SCAI classification. If validated in larger cohorts, the SAVE ACS classification could form the substrate of future randomized trials of early MCS in ACS CS patients.

## Figures and Tables

**Table 1:** Independent predictors (categorised) of 30-day mortality.

Variables	OR	95% CI	*p* value
Age (per 10 years increase)	1.69	1.41 to 2.03	<0.001
SBP <90 mmHg	2.28	1.35 to 3.85	0.002
Ventilated on arrival	6.44	3.72 to 11.17	<0.001
LVEF			
Normal/mildly impaired	1		
Moderately impaired	1.95	1.15 to 3.29	0.013
Severely impaired	2.3	1.33 to 3.97	0.003
Lactate (mmol/L)*∗*			
<2	1		
2–5	1.81	1.09 to 3.02	0.023
>5	7.2	3.63 to 14.34	<0.001
Base excess tertiles (mmol/L)			
−0.4 to +16	1		
−3.9 to −0.5	3.67	1.66 to 8.12	0.001
−28 to −4	4.51	2.02 to 10.07	<0.001

Model AUC 0.883, (0.856 to 0.911), *p* < 0.001 for death in 30 days and OR: odds ratio, CI: confidence interval, SBP: systolic blood pressure, LVEF: left ventricular ejection fraction, AUC: area under the curve, *∗*the SCAI shock classification lactate cutoffs were used.

**Table 2:** Frontline SAVE ACS classification.

	No shock	A	B	C	D	E
N (%)	163 (17.5%)	98 (10.4%)	287 (30.4%)	262 (27.7%)	109 (11.5%)	24 (2.5%)
SBP	≥90 mmHg	≥90 mmHg	Any	Any	Any	<90 mmHg
LV impairment	Mild	Mod/severe	Mod/severe	Mod/severe	Mod/severe	Severe
Lactate	<2	<2	<2	2–5	>5	>5
BE	>−0.5	>−0.5	<−0.5	<−0.5	<−0.5	<−4
30-day mortality (%)	1.2	4.1	13.6	29.4	64.2	87.5
30-day mortality (%) if not intubated	0.7	2.3	11.2	23.4	55.6	62.5

Data from 943 patients classified according to the SAVE criteria, AUC for 30-day mortality 0.814 (0.782 to 0.845), *p* < 0.001, SBP: systolic blood pressure, LV: left ventricle, BE: base excess, and AUC: area under the curve.

**Table 3:** Forward conditional logistic regression analysis determining main independent “frontline” predictors of 30-day mortality.

		OR (95% CI)	*p* value	AUC _*C*-statistic_	*p* value
Model 1	Lactate	1.55 (1.42 to 1.69)	<0.001	0.787 (0.745 to 0.828)	<0.001
Model 2	Lactate	1.46 (1.34 to 1.6)	<0.001	0.830*∗*	<0.001
	Intubated	6.09 (3.9 to 9.6)	<0.001	0.793 to 0.867)	

Model 3	Lactate	1.48 (1.35 to 1.62)	<0.001	0.858*∗* (0.825 to 0.891)	<0.001
	Intubated	9.74 (5.8 to 16.33)	<0.001		
	Age	1.06 (1.04 to 1.08)	<0.001		

Model 4	Lactate	1.28 (1.15 to 1.43)	<0.001	0.868*∗* (0.837 to 0.900)	<0.001
	Intubated	9.35 (5.55 to 15.74)	<0.001		
	Age	1.06 (1.04 to 1.08)	<0.001		
	BE	1.11 (1.05 to 1.17)	<0.001		

Model 5	Lactate	1.27 (1.13 to 1.42)	<0.001	0.878 (0.850 to 0.907)	<0.001
	Intubated	78.34 (4.89 to 14.2)	<0.001		
	Age	1.05 (1.04 to 1.07)	<0.001		
	BE	1.11 (1.05 to 1.17)	<0.001		
	LVEF-mild/*N*	REF			
	LVEF-mod	1.88 (1.21 to 3.16)	0.017		
	LVEF-severe	2.18 (1.28 to 3.75)	0.004		

Model 6	Lactate	1.25(1.12 to 1.41)	<0.001	0.879 (0.851 to 0.908)	<0.001
	Intubated	7.4 (4.3 to 12.74)	<0.001		
	Age	1.05 (1.04 to 1.07)	<0.001		
	BE	1.10 (1.04 to 1.17)	<0.001		
	LVEF-mild/*N*	REF			
	LVEF-mod	1.84 (1.09 to 3.1)	0.022		
	LVEF-severe	1.98 (1.14 to 3.42)	0.015		
	SBP	0.99 (0.982 to 0.999)	0.024		

*N* = 892 patients included in the analysis. J: variables included in the regression model: age, gender, cardiac arrest, ongoing resuscitation on admission, ECG on admission, intubated/ventilated on admission, LVEF category, SBP, pH, lactate, and BE, *∗*statistically significant increase in the AUC when compared to previous model, OR: odds ratio, CI: confidence interval, AUC: area under the curve, BE: base excess, LVEF: left ventricular ejection fraction, SBP: systolic blood pressure, and ECG: electrocardiogram.

**Table 4:** Frontline variables in ACS patients grouped by vital status at 30 days.

	Survival at 30 days		*p* value	AUC
	Alive (*N* = 10548)	Deceased (*N* = 891)		
Demographics (*N* = 11439)				
Age (years)	63.4 ± 14.2	72.6 ± 14.0	<0.001	
Gender (male) *N* (%)	7687 (72.9)	593 (66.6%)	<0.001	
Clinical assessment (*N* = 10107)				
Killip class (%)				0.675 (0.651–0.698) ^*∗*^
(I) No evidence of heart failure	91.5	58.1	<0.001	
(II) Basal crepitations/raised venous pressure	4.5	9.2		
(III) Pulmonary oedema	2.6	8.8		
(IV) Cardiogenic shock	1.4	23.9		
Baseline blood pressure (*N* = 6110)				
Systolic blood pressure (mmHg)	129.2 ± 25.2	98 ± 36.2	<0.001	0.770 (0.742–0.798) ^*∗*^
Diastolic blood pressure (mmHg)	77.9 ± 16.9	79.2 ± 29.8	0.411	
Inotropic support on arrival *N* (%)	72 (0.7)	57 (6.4)	<0.001	
Electrocardiogram on admission (*N* = 11189) (%)				
No dynamic changes	10.6	7.7	<0.001	
LBBB	4.9	6.1		
Other abnormality	5.2	9.7		
T-wave changes	7.4	2.6		
ST depression	9	11.7		
ST segment elevation	63	62.2		
Bedside LV systolic function assessment (*N* = 4448) (%)				
Good/mildly impaired (>45%)	70.3	30.7	<0.001	
Moderate impairment (35–45%)	20.4	27.6		0.728 (0.700–0.756) ^*∗*^
Severely impaired (<35%)	9.3	41.7		
Arterial blood gas on admission (*N* = 3752)				
Lactate (mmol/L)	1.5 (1.1 to 2.4)	3.7 (2.0 to 7.1)	<0.001	0.780 (0.759–0.801) ^*∗*^
pH	7.41 (7.37 to 7.45)	7.31 (7.24 to 7.39)	<0.001	0.773 (0.743–0.803) ^*∗*^
pO2 (kPa)	11.3 (9.11 to 14.2)	12.55 (9.35 to 21.45)	<0.001	0.559 (0.532–0.586) ^*∗*^
pCO2 (kPa)	4.92 (4.35 to 5.5)	5.29 (4.37 to 6.27)	<0.001	0.580 (0.552–0.609) ^*∗*^
BE (mmol/L)	−1.2 (−3.6 to 1)	−6.35 (−8.9 to 2.8)	<0.001	0.778 (0.756–0.800) ^*∗*^
K (mmol/L)	3.8 (3.5 to 4.2)	4.1 (3.7 to 4.6)	<0.001	0.605 (0.578–0.632) ^*∗*^
Glc (mmol/L)	7.6 (6.3 to 10.1)	10.6 (7.78 to 14.8)	<0.001	0.693 (0.670–0.717) ^*∗*^
Cardiac arrest parameters (*N* = 11439)				
Cardiac arrest *N* (%)	816 (7.7)	458 (51.4)	<0.001	
Cardiac arrest on admission—ongoing resuscitation *N* (%)	6 (0.1)	29 (3.3)	<0.001	
Ventilated preprocedure *N* (%)	215 (2.1)	273 (32.1)	<0.001	
Type of arrest			<0.001	
Asystole	8.2	18.2		
EMD	2.8	21.1		
VF/pulseless VT	79.2	58.1		
Unknown	9.8	2.6		

AUC: area under the curve, LBBB: left bundle branch block, LV: left ventricle, BE: base excess, Glc: glucose, EMD: electromechanical dissociation, VF: ventricular fibrillation, and VT: ventricular tachycardia.

**Table 5:** Past medical history and procedural data grouped by vital status at 30 days.

	Survival at 30 days		*p* value
	Alive (*N* = 10548)	Deceased (*N* = 891)	
Past medical history			
Previous MI *N* (%)	1679 (16.1)	163 (18.7)	0.045
Previous CABG *n* (%)	553 (5.3)	80 (9.2)	<0.001
Previous PCI *N* (%)	1311 (12.6)	83 (9.5)	0.008
Diabetes *N* (%)	2020 (19.9)	198 (23.5)	0.014
Hypertension *N* (%)	4347 (41.2)	344 (38.6)	0.129
Total cholesterol	5.34 ± 16.2	5.1 ± 1.5	0.916
Asthma/COPD *N* (%)	682 (6.6)	88 (10.4)	<0.001
CVA *N* (%)	85 (0.8)	12 (1.3)	0.091
Creatinine	87.5 ± 1.36	131.3 ± 5.49	<0.001
Smoking status (%)			
Never smoked	43.4	48.5	<0.001
Ex-smoker	27.4	31.2	
Current smoker	29.1	20.3	
Procedural data			
LMS disease *N* (%)	301 (4.4)	93 (20)	<0.001
Epicardial disease *N* (%)			
Single-vessel	4226 (63.2)	195 (44.6)	<0.001
Two-vessel	1632 (24.4)	138 (31.6)	
Three-vessel	626 (9.4)	95 (21.7)	
Inotropes *N* (%)	72 (0.7)	57 (6.4)	<0.001
IABP n(%)	213 (2)	96 (10.8)	<0.001
Impella*∗N* (%)	7 (0.1)	9 (1)	<0.001
ECMO*∗N* (%)	8 (0.1)	20 (2.2)	<0.001
TIMI flow after PCI (%)			
0	2.1	7.7	<0.001
I	0.9	4.1	
II	3.7	12.3	
III	93.2	75.9	
Access site (%)			
Radial	63.9	35.9	<0.001
Femoral	36.1	64.1	

*∗*ECMO and Impella entry in database 2018 onwards, MI: myocardial infarction, CABG: coronary artery bypass surgery, PCI: percutaneous coronary intervention, COPD, chronic obstructive pulmonary disease, CVA: cerebrovascular accident, LMS: left main stem disease, IABP: intra-aortic balloon pump, ECMO: extra corporeal membrane oxygenation, and TIMI: thrombolysis in myocardial infarction.

## Data Availability

Data are available from the corresponding author upon request.

## Supplementary Materials

Supplementary Materials
*Supplementary Table 1.* Cox-regression analysis for long-term all-cause mortality. *Supplementary Figure*. Relative contraindications to mechanical circulatory support.
