# Supplementary material for: Rapid Classification and Treatment Algorithm of Cardiogenic Shock Complicating Acute Coronary Syndromes: The SAVE ACS Classification
Source: J Interv Cardiol. 2022 Jan 12;2022:9948515. doi: 10.1155/2022/9948515 (PMC8769867; doi:10.1155/2022/9948515)
Supplement: Supplementary Materials — Supplementary Table 1. Cox-regression analysis for long-term all-cause mortality. Supplementary Figure. Relative contraindications to mechanical circulatory support. [file 9948515.f1.docx]

| **Suppl. Table 1**. Cox regression analysis for long-term all cause mortality | | | |
| --- | --- | --- | --- |
| **Variable** | **HR** | **95% CI** | **P-value** |
| Age (per year) | 1.06 | 1.04 to 1.07 | <0.001 |
| Lactate | 1.12 | 1.05 to 1.18 | <0.001 |
| Ventilated on arrival | 3.22 | 1.81 to 5.72 | <0.001 |
| LVEF  Normal/mildly impaired  Moderately impaired  Severely impaired | 1  1.74  2.4 | 1.23 to 2.45  1.67 to 3.40 | 0.002  <0.001 |
| BE | 1.06 | 1.02 to 1.1 | 0.002 |
| SBP | 1 | 0.99 to 1.004 | 0.506 |
| HR: hazard ratio, CI: confidence interval, LVEF: left ventricular ejection fraction, BE: base excess, SBP: systolic blood pressure | | | |

**Supplementary Figure.** Relative contraindications to mechanical circulatory support
